# Cohesin without Cohesion: A Novel Role for Pds5 in *Saccharomyces cerevisiae*

**DOI:** 10.1371/journal.pone.0100470

**Published:** 2014-06-25

**Authors:** Kevin Tong, Robert V. Skibbens

**Affiliations:** Department of Biological Sciences, Lehigh University, Bethlehem, Pennsylvania, United States of America; National Cancer Institute, United States of America

## Abstract

High fidelity chromosome segregation during mitosis requires that cells identify the products of DNA replication during S-phase and then maintain that identity until anaphase onset. Sister chromatid identity is achieved through cohesin complexes (Smc1, Smc3, and Mcd1 and Irr1/Scc3), but the structure through which cohesins perform this task remains enigmatic. In the absence of unambiguous data, a popular model is that a subset of cohesin subunits form a huge ring-like structure that embraces both sister chromatids. This ‘*one-ring two-sister chromatid embrace*’ model makes clear predictions – including that premature cohesion loss in mitotic cells must occur through a substantial reduction in cohesin-DNA associations. We used chromatin immunoprecipitation to directly test for cohesin dissociation from well-established cohesin binding sites in mitotic cells inactivated for Pds5 – a key cohesin regulatory protein. The results reveal little if any chromatin dissociation from cohesins, despite a regimen that produces both massive loss of sister chromatid tethering and cell inviability. We further excluded models that cohesion loss in mitotic cells inactivated for Pds5 arises through either cohesin subunit degradation, premature Hos1-dependent Smc3 de-acetylation or Rad61/WAPL-dependent regulation of cohesin dynamics. In combination, our findings support a model that cohesin complexes associate with each sister and that sister chromatid cohesion likely results from cohesin-cohesin interactions. We further assessed the role that Pds5 plays in cohesion establishment during S-phase. The results show that Pds5 inactivation can result in establishment defects despite normal cohesion loading and Smc3 acetylation, revealing a novel establishment role for Pds5 that is independent of these processes. The combination of findings provides important new insights that significantly impact current models of both cohesion establishment reactions and maintenance.

## Introduction

Survival at the cellular level and proper development at the organismal level require that cells accurately replicate DNA during S phase and then properly segregate the resulting sister chromatids into each of the daughter cells during mitosis. To ensure high fidelity chromosome segregation, the products of DNA replication must be identified as sisters and that identity maintained until anaphase onset. Identity is achieved through cohesin complexes (Smc1, Smc3, and Mcd1/Scc1/RAD21 and Irr1/Scc3/SA1,2) which tether together sister chromatids. The cohesin structure through which sister chromatids are tethered together remains unknown, but current models of cohesin-DNA interactions include that DNA resides within the lumen of huge bi-partite or tri-partite rings, is held within a C-clamp configuration, or that DNA is sandwiched between SMC head domains and an Mcd1 capping structure [1].

Cohesins are regulated by the coordinated activity of numerous accessory factors to achieve cohesion. For instance, cohesins are loaded onto DNA at specific cohesin-associated regions (CARs) by the Scc2 (NIPBL) and Scc4 (MAU2) heterocomplex [2]–[6]. Cohesin deposition onto DNA occurs throughout a major portion of the cell cycle (providing for cohesin function in transcription, DNA repair, DNA replication, chromosome segregation and condensation), but deposition during S-phase is essential for cohesion [1]. Scc2 association with DNA requires Chl1 DNA helicase. Chl1 functions during S-phase and interacts with the Okazaki maturation factor Fen1, further supporting the model that cohesin deposition occurs in the context of replicated sister chromatids [7], [8]. Cohesin binding to DNA, however, is not sufficient to tether together sister chromatids. Instead, chromatin-bound cohesins must be converted to a tethering competent state by the Ctf7/Eco1/EFO1,2/ESCO1,2 family of acetyltransferases which modify evolutionarily conserved lysines on Smc3 [9]–[16]. Eco1/Ctf7-dependent cohesion establishment is essential during S-phase, consistent with numerous interactions between Eco1/Ctf7 and DNA replication factors [12], [13], [17]. A recent study also reveals histone variant H2A.Z as an accessory factor in cohesion [18]. In combination, these studies provide compelling evidence that cohesion deposition and establishment are temporally coordinated and occur in concert with chromatin assembly reactions that occur on newly replicated DNA [1], [19].

Additional cohesin-auxiliary factors impact cohesin association with DNA. For instance, Rad61/WAPL appears to promote cohesin dissociation such that *rad61/wapl* mutation results in unresolved and hypercondensed sister chromatids [20]–[23]. Based on findings that *rad61/wapl* deletion bypasses *eco1/ctf7* mutant cell inviability (leading to the notion that Rad61/WAPL is an anti-establishment factor), a model forwarded was that Eco1/Ctf7-dependent Smc3 acetylation displaces Rad61/WAPL to promote stable cohesin binding to DNA [24], [25]. Careful analyses, however, reveal that *rad61/wapl* deletion rescues *eco1/ctf7* mutant cell chromatin condensation defects – not cohesion defects [26]. Thus, the mechanism through which Eco1/Ctf7-dependent acetylation of Smc3 drives cohesion establishment (and condensation) remains enigmatic. What is clear is that Smc3 must return to a de-acetylated state prior to the next cell cycle – a process mediated by Hos1/HDAC8 [27]–[30].

Pds5 is a particularly intriguing cohesin-auxiliary protein that highlights the complexity of both establishment reactions and cohesion maintenance. Early findings, in part predicated on *pds5-1* and *pds5-101* alleles, document that Pds5 both binds cohesins and is required for the maintenance of cohesion during mitosis [31]–[33]. In contrast, *pds5-99* mutant cells maintain cohesion once established, but appear deficient in cohesin loading (or retention) onto DNA [32]. A mechanism through which Pds5 may impact Scc2,Scc4-dependent cohesin deposition remains unknown. Pds5 also binds Rad61/WAPL and Irr1/Scc3 [24], [25], [34], in support of the notion that Pds5 promotes both stable cohesin-DNA association and chromatin condensation [26]. It is thus notable that Pds5 is critical for chromosome condensation, attributes shared by both Eco1 and Mcd1 [12], [31], [35]. Pds5 also binds Eco1/Ctf7 in vitro and promotes Eco1/Ctf7-dependent acetylation of Smc3 in vivo [36]–[38], in support of numerous studies that suggest that cohesin deposition and cohesion establishment are temporally coordinated [17]. Intriguingly, while *pds5-1* is lethal in combination with *eco1/ctf7* alleles [37], certain other *pds5* alleles bypass a requirement for Eco1/Ctf7, even though these *pds5 eco1/ctf7* double mutant cells exhibit significant cohesion defects [24]–[26]. The extent through which this rescue involves condensation pathways, similar to *rad61/wapl eco1/ctf7* double mutant cells, remains an untested but intriguing possibility [26]. Given this surplus of roles, the confusion regarding which activity (cohesin deposition, cohesion anti-establishment, cohesion maintenance, or chromosome condensation) comprises the essential function of Pds5 is not surprising. Since *PDS5/APRIN* mutations arise in both cancer progression and developmental abnormalities [39]–[42], resolving these issues remains of significant clinical interest. Here, we characterize a particularly instructive separation-of-function allele of *PDS5* that challenges current paradigms in cohesion maintenance and establishment.

## Results

### Pds5 is essential for cell viability and cohesion maintenance specifically during mitosis

**Figure 1:**
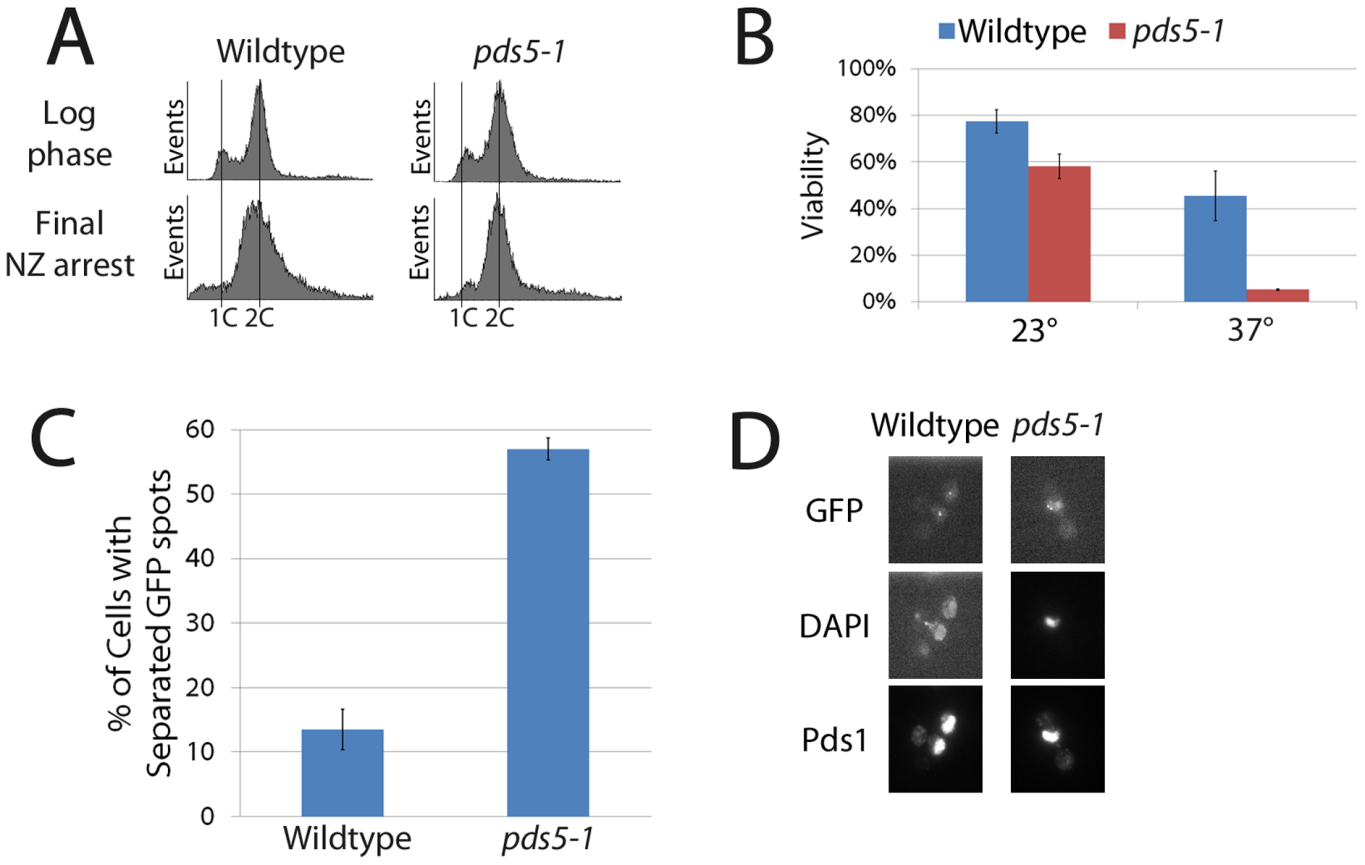
Pds5 is essential for cohesion maintenance. (A) Flow cytometry analyses revealing DNA content of wildtype and *pds5-1* mutant cells prior to and following 3 hour incubation in nocodazole (cultures were shifted to the restrictive temperature during the final hour of incubation in medium supplemented with nocodazole). (B) Percent viability of wildtype and *pds5-1* mutant cells in the presence or absence of the final shift to the restrictive temperature during mitotic arrest. (C) Percent cohesion defects of wildtype and *pds5-1* mutant cells after incubation at non-permissive temperature as described in (A) above (D) Micrographs of wildtype and *pds5-1* mutant cells showing separated sisters (GFP-TetR), DNA (DAPI) and retention of Pds1 indicative of a pre-anaphase state.

Despite the essential role that Pds5 plays in budding yeast, its role in cohesion maintenance remains unknown. Using the temperature sensitive allele *pds5-1,* we first confirmed that Pds5 is essential to retain cell viability and maintain sister chromatid cohesion during an extended metaphase arrest. Wildtype and *pds5-1* mutant cells were synchronized in pre-anaphase at a temperature permissive for *pds5*-1 mutant strains and then shifted to a temperature restrictive for pds5-1 protein, while maintaining the mitotic arrest, to limit inactivation to an extended pre-anaphase (Figure 1A). Cells were then plated onto rich medium plates at the permissive temperature and viability analyzed by colony growth assays. Wildtype cells exhibit 45% viability after incubation at the non-permissive temperature, consistent with prior studies that this regimen is stressful even to wildtype cells, but that a significant fraction of cells remain viable [31]. In contrast, *pds5-1* mutant cells are predominantly inviable, exhibiting only 4% colony growth (Figure 1B). We next tested whether *pds5-1* mutant cells indeed exhibit cohesion defects upon inactivation specifically during mitosis using a cohesion assay strain in which a *TetO* array, integrated approximately 40 kb from centromere V, is detected through the binding of GFP-tagged *TetR* protein [43], [44]. This cohesion assay strain also contains epitope-tagged Pds1p (an inhibitor of anaphase onset) so that pre-anaphase cells can be unambiguously identified [45]. Quantification of GFP signals reveal that wildtype pre-anaphase cells show very low levels (∼10%) of premature sister chromatid separation. In contrast, *pds5-1* mutant cells exhibit a significant level (∼55%) of cohesion defects during pre-anaphase (Figure 1C and D), a level identical to that previously reported for this allele [31]. In combination, the above results confirm that Pds5 is both essential to retain cell viability and required to maintain sister chromatid cohesion specifically during an extended metaphase arrest [31]–[33].

### Cohesin enrichment to DNA is retained in cohesion defective *pds5-1* mutant cells during mitosis

**Figure 2:**
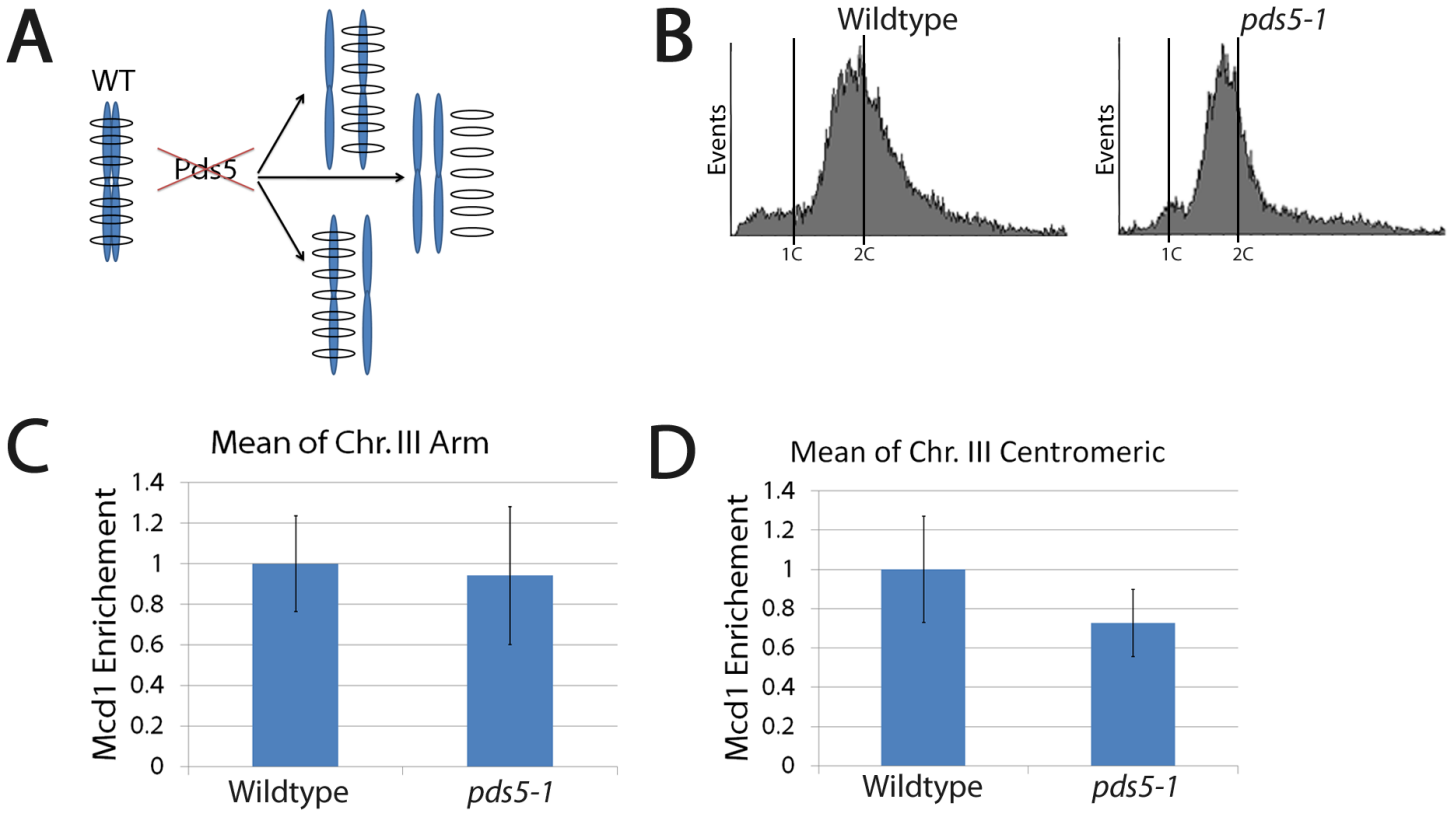
Inactivation of Pds5 during mitosis results in cohesion loss in the absence of cohesin dissociation from DNA. (A) Schematic highlights possible mechanisms through which cohesion loss may occur in the 1-ring two sister chromatids embrace model. See text for details. (B) DNA content of wildtype and *pds5-1* mutant cells treated as described in Figure 1A. (C and D) Mcd1 enrichment along arm and pericentromeric CAR sites shown are averages of three independent experiments obtained from wildtype (normalized to 1) and *pds5-1* mutant cells.

What is the mechanism through which Pds5 inactivation, specifically during mitosis, produces cohesion defects? For the *one-ring two-sister chromatid embrace* model (in which sister chromatids A and A′ are embraced by a cohesin ring), cohesion loss can only occur through one of three possible reactions: either chromatid A exits the ring (A′ is retained), chromatid A′ exits the ring (A is retained), or both A and A′ exit from the ring. If each of the three outcomes occurs with equal probability within a population, then cohesin enrichment onto DNA should drop to approximately 33% in cohesion deficient cells compared to cells that retain cohesion (Figure 2A). To test this prediction, wildtype and *pds5-1* mutant cells both expressing MYC-tagged Mcd1 were synchronized in pre-anaphase at the permissive temperature, shifted to the restrictive temperature while maintaining the mitotic arrest (Figure 2B), then subjected to chromatin-immunoprecipitation (ChIP) to assess Mcd1 association with chromatin at 13 loci comprising several well-documented Cohesin-Associated Regions (CAR) along chromosome arm and pericentromeric regions of chromosome III (see below). We first analyzed the data *en masse* to approximate a genome-wide role for Pds5 in cohesin retention onto DNA. The results show that *pds5-1* mutant cells exhibit 95% of cohesin binding along chromosome arm CARs compared to wildtype cells (Figure 2C). *pds5-1* mutant cells also exhibit cohesin binding along the pericentromeric domain that was only marginally lower (∼75%) than that observed for wildtype cells (Figure 2D).

**Figure 3:**
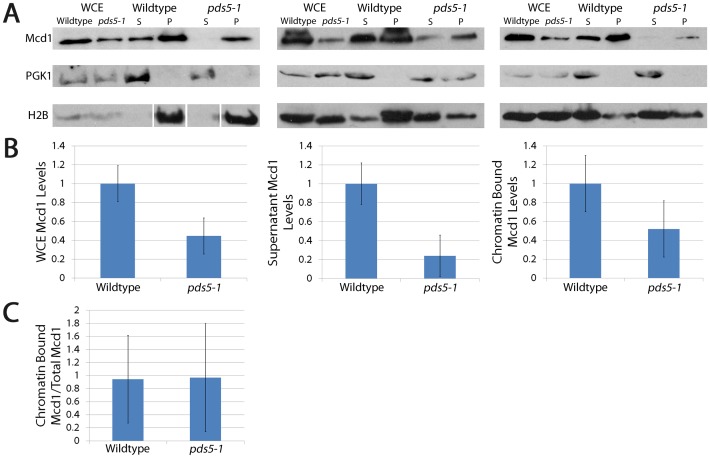
*pds5-1* mutant cells exhibit reduced Mcd1 levels but retain high levels of Mcd1 cohesin enrichment to DNA. (A) Triton X-100 fractionation assays of wildtype and *pds5-1* mutant cells expressing Mcd1-MYC. Western blots performed on the resulting whole cell extracts (WCE), soluble fractions (S) and chromatin-bound pelleted fractions (P). Histone 2B (H2B) and Phosphoglycerate kinase (PGK) serve as controls for soluble and chromatin-bound proteins, respectively. Results shown for three independent fractionation studies (Mcd1 in wildtype normalized to 1). (B) Quantifications of Mcd1 in whole cell extracts, supernatants, and chromatin pellet fractions. Mcd1 enrichment to DNA is based on the ratio of Mcd1 to Histone 2B levels obtained from 3 independent experiments while the soluble pool of Mcd1 is based on the ratio of Mcd1 to PGK levels from 3 independent experiments. Mcd1 levels in wildtype cells normalized to 1. (C) Ratio of chromatin bound Mcd1 in pellet to total levels in whole cell extracts (normalizing to H2B) reveal that equivalent proportions of Mcd1 remains chromatin bound in both wildtype and *pds5-1* mutant cells.

We decided to independently assess the global retention of cohesin in *pds5-1* mutant cells using Triton X-100 cell fractionation assays, a documented procedure previously used to demonstrate chromatin-associations of cohesin and other factors [8], [13]. Log phase wildtype and *pds5-1* mutant cells held at the permissive temperature in medium supplemented with nocodazole to arrest cells pre-anaphase were shifted to the non-permissive temperature while maintaining the mitotic arrest, harvested, lysed and then processed for fractionation analysis. Fractionation of whole cell lysate into soluble and chromatin-associated components was confirmed using Phosphoglycerokinase (PGK) as a cytosolic marker and Histone 2B (H2B) as a chromatin marker, as previously described [8]. We then assessed fractionation of Mcd1, a core subunit of the cohesin complex, to the chromatin pellet and compared these values to Histone 2B loading control levels. We also assessed Mcd1 fractionation into the soluble pool, using PGK levels as our loading control. Western blot results are shown for each of three independent experiments (Figure 3A). Quantifications of soluble and chromatin-associated Mcd1 are provided as averages from these 3 independent experiments with the level of Mcd1 in *pds5*-1 mutant cells compared to the level of Mcd1 observed in wildtype cells (Figure 3B). Intriguingly, *pds5-1* mutant cells exhibit Mcd1 levels in whole cell lysates that are significantly lower than the level of Mcd1 in whole cell lysates from wildtype cells (Figure 3B, left panel). Importantly, however, further analyses of fractionated components reveal that the reduction in Mcd1 levels occurs predominantly in the soluble pool (compare Mcd1 levels in *pds5-1* mutant cells in left panel to that in middle right panel of Figure 3B). In contrast, Mcd1 levels in the chromatin fraction are nearly identical to that present in whole cell extracts from *pds5-1* mutant cells (compare Mcd1 levels in *pds5-1* mutant cells left panel to that in right panel of Figure 3B). To quantify this further, we compared the level of chromatin-bound Mcd1 to that present in the whole cell lysates for both wildtype and *pds5-1* mutant cells. The results show that *pds5-1* mutant cells are equally competent as wildtype cells in cohesin enrichment to DNA (Figure 3C). In combination, these results reveal that Mcd1 levels are reduced in pre-anaphase *pds5-1* mutant cells held at the restrictive temperature, relative to wildtype cells, but that cohesin retention onto DNA is fully retained in *pds5-1* mutant cells. Thus, bulk cohesin-dissociation from DNA is not the basis for the cohesion defects that occur in *pds5-1* mutant cells.

**Figure 4:**
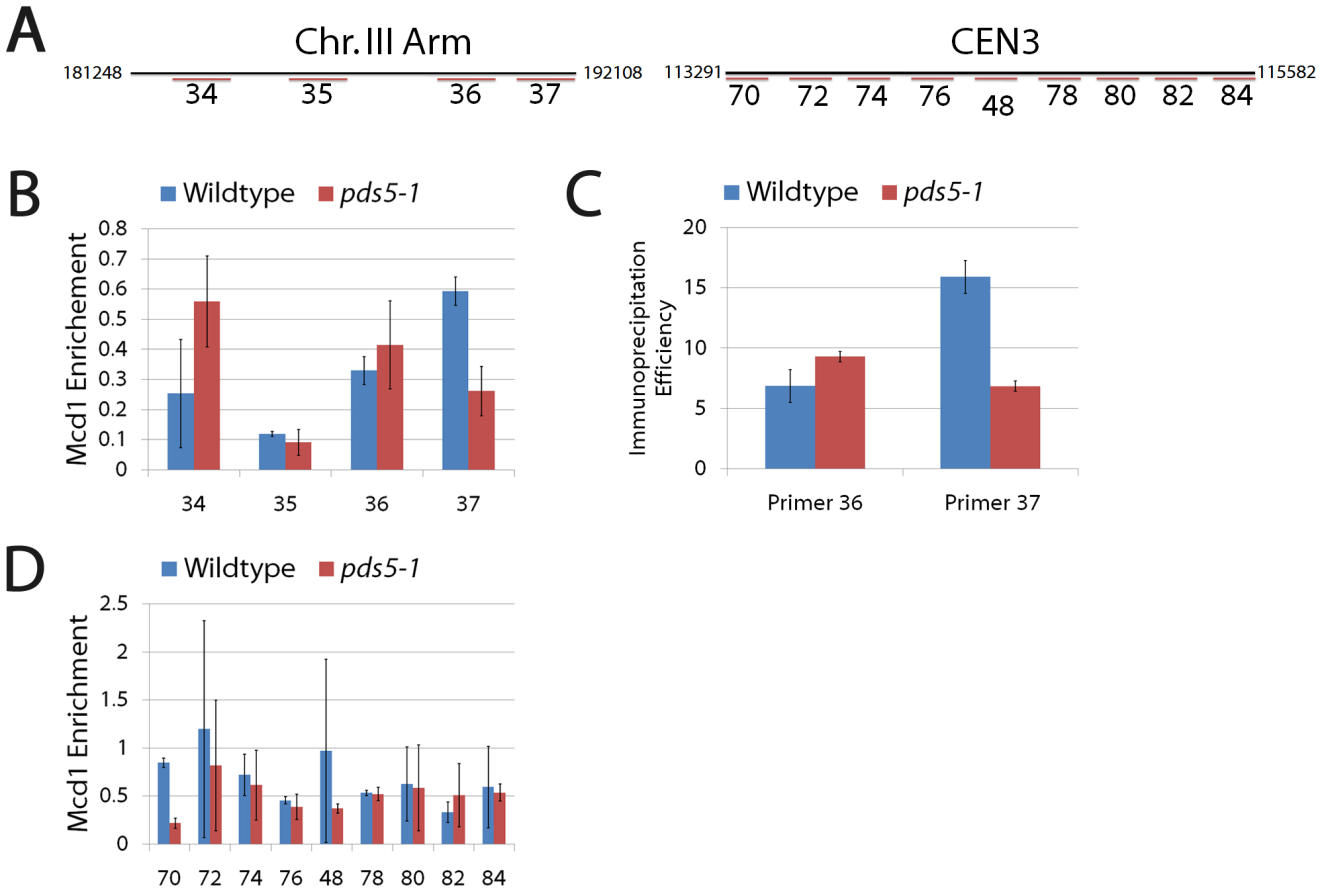
Sister chromatid cohesion loss occurs despite retention of cohesin enrichment along chromosome arm and pericentromeric CAR sites. (A) Position of primers used in ChIP along individual arm (comprising two CAR sites) and pericentromeric CAR sites for chromosome III. (B) Mcd1 enrichment along chromosome arm CARs are averages of three independent experiments obtained using four oligo pairs (34, 35, 36 and 37) in wildtype and *pds5-1* mutant cells. (C) Immunoprecipitation efficiency obtained using Quantitative PCR performed on CAR sites 36 and 37 confirm the Mcd1 enrichment levels observed using ChIP in both wildtype and *pds5-1* mutant cells. (D) Mcd1 enrichment along pericentromeric CARs obtained from nine oligo pairs (70, 72, 74, 76, 48, 78, 80, 82 and 84) in wildtype and *pds5-1* mutant cells. All primer design and designations from [5], [72].

We next assessed whether Pds5 inactivation adversely impacts cohesin enrichment to specific loci comprising well-documented CARs (Figure 4A). We first turned to individual chromosome arm CARs, performing ChIPs on lysates obtained for wildtype and *pds5-1* mutant cells maintained at a permissive temperature in medium supplemented with nocodazole to arrest cells in pre-anaphase and then shifting to the restrictive temperature to inactive pds5-1 protein specifically during the pre-anaphase arrest. The results show that *pds5-1* mutant cells overall exhibit levels of cohesin enrichment onto DNA that are nearly identical to those observed in wildtype cells, despite the loss of cohesion in the *pds5-1* mutant cells. Careful analyses revealed, however, that cohesin enrichment varies for given loci. Among four individual arm sites comprising two CARs, three exhibit either equivalent (35) or elevated cohesin enrichment (34 and 36) in *pds5-1* mutant cells compared to wildtype cells (Figure 4B). Conversely, only one site (37) exhibits a reduction (40%) in cohesin enrichment in *pds5-1* mutant cells compared to wildtype cells (Figure 4B). Both the increase and decrease of cohesin enrichment in *pds5-1* mutant cells, compared to wildtype cells, was intriguing. Thus, we decided to independently test for cohesin enrichment onto DNA at selected loci using quantitative PCR (Figure 4C). Results from qPCR reveal that *pds5-1* mutant cells indeed contain elevated levels of cohesin enrichment at site 36 but contain less cohesin enrichment at site 37 compared to wildtype cells that retain cohesion, confirming results obtained through ChIP.

Does cohesin enrichment remain elevated along the centromere in *pds5-1* mutant cells in which cohesion is abolished? To address this question, we performed similar analyses on nine individual sites that comprise the pericentromeric domain of chromosome III. Of the nine sites assayed, six sites (72, 74, 76, 78, 80, 84) retain cohesin enrichment to DNA in *pds5-1* mutant cells at levels nearly identical to that of wildtype cells (Figure 4D). One site (82) exhibited slightly elevated levels of cohesin-enrichment in *pds5-1* mutant cells, relative to wildtype cells. Only in the remaining two sites (70 and 48) did we find that cohesin enrichment in *pds5-1* mutant cells is reduced (25% and 40% respectively) relative to wildtype cells. Each CAR site was validated using *scc2-4* mutant cells (see below). The combined results from both chromosome arm and pericentromeric ChIP studies reveal that the cohesion loss that occurs upon Pds5 inactivation during mitosis does so despite levels of chromatin-bound cohesins that are similar to wildtype cells, but that variation in cohesin enrichment occurs within a limited number of specific loci.

### Cohesin acetylation is retained in cohesion defective *pds5* mutant cells during mitosis

**Figure 5:**
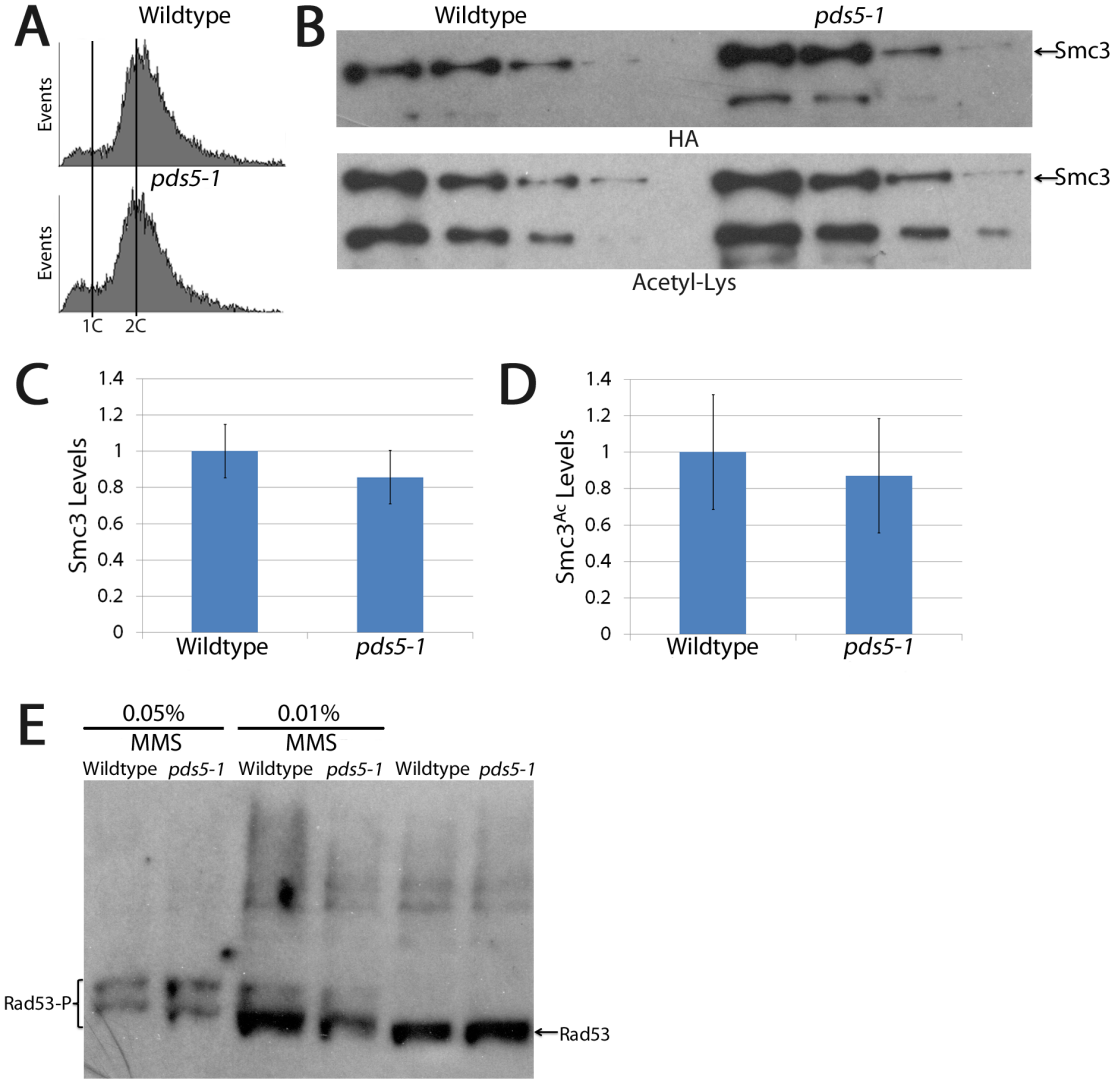
Sister chromatid cohesion loss occurs despite retention of Smc3 acetylation and in the absence of DNA damage. (A) DNA content of wildtype and *pds5-1* mutant cells treated as described in Figure 1A. (B) Dilution series of Smc3 immunoprecipitated from wildtype and *pds5-1* mutant cells revealing total Smc3 protein (HA) and acetylation (Acetyl-Lys) levels. (C and D) Quantification of total Smc3 protein and Smc3 acetylation levels in wildtype (normalized to 1) and *pds5-1* mutant cells. (E) Wildtype and *pds5-1* mutant cells are competent to phosphorylate Rad53 in response to DNA damage (MMS), but do not phosphorylate Rad53 in the absence of MMS.

The above findings that cohesin enrichment to DNA is retained in cohesion-deficient *pds5-1* mutant cells suggest that ring opening and chromatid release is not the mechanism through which sister chromatids separate. We realized, however, that the above analyses do not exclude the possibility that the chromatin-associated cohesins detected are newly deposited. Eco1/Ctf7 acetylates Smc3 only during S-phase, a modification temporally limited to S-phase by Eco1/Ctf7 phosphorylation (by Cdk1), ubiquitination (by Cdc4/SCF) and degradation upon entry into G2 [11], [12], [14], [16], [46]–[48]. If the chromatin-associated cohesins that we detect in mitotic *pds5-1* mutant cells are newly (mitotically) deposited, then those cohesins should be devoid of acetylated Smc3. To test which population of cohesins persist in mitotic but cohesion-deficient *pds5-1* mutant cells, log phase wildtype and *pds5-1* mutant cells expressing HA-tagged Smc3 were synchronized in pre-anaphase, shifted to non-permissive temperature while maintaining the pre-anaphase arrest (Figure 5A), and normalized cell densities lysed and incubated with anti-HA coupled affinity matrix. After washing to remove unbound or weakly associated proteins, Smc3 protein was eluted from the beads and assayed by Western blot. A dilution series confirmed that sample concentrations provide for linear range signal detection (Figure 5B). Smc3 levels in *pds5-1* mutant cells were compared to those observed in wildtype cells – average values shown from three different experiments (Figure 5C). Importantly, quantitative analyses from these dilution series reveal that total Smc3 protein levels in *pds5-1* mutant cells are similar (85%) to that of wildtype cells (Figures 5C), indicating that Smc3 and Mcd1 levels are regulated through different pathways. The same blot was then reprobed (after confirming signal removal) to assess the level of Smc3 acetylation. The results reveal that 85% of Smc3 is acetylated in *pds5-1* mutant cells, compared to wildtype (Figure 5D), consistent with the model that the majority of Smc3 exists in an acetylated state that is attained during S-phase.

Despite Eco1/Ctf7 degradation upon exit from S-phase, we were concerned that *pds5-1* protein inactivation might produce DNA damage during G2 that could in turn induce a new wave of Eco1/Ctf7 establishment activity [47]–[49]. We therefore decided to test whether pds5-1 protein inactivation induces DNA damage, which would promote Eco1/Ctf7 re-establishment. We first confirmed that both wildtype and *pds5-1* mutant cells are competent to respond to DNA damage after exposure to methyl methanesulfanate (MMS). Importantly, neither mitotic wildtype or *pds5-1* mutant cells shifted to the restrictive temperature in the absence of MMS result in Rad53 phosphorylation (Figure 5E), negating the model that Eco1/Ctf7 becomes reactivated during G2/M in response to pds5-1 protein inactivation. In combination, these findings reveal that the acetylated DNA-enriched cohesins present in *pds5-1* mutant cells are the product of Eco1/Ctf7-dependent cohesion establishment reactions that occur during S-phase, not by mitotic loading or subsequent DNA damage-induced response by Eco1.

### Pds5 role in cohesion maintenance occurs independent of Rad61/WAPL

**Figure 6:**
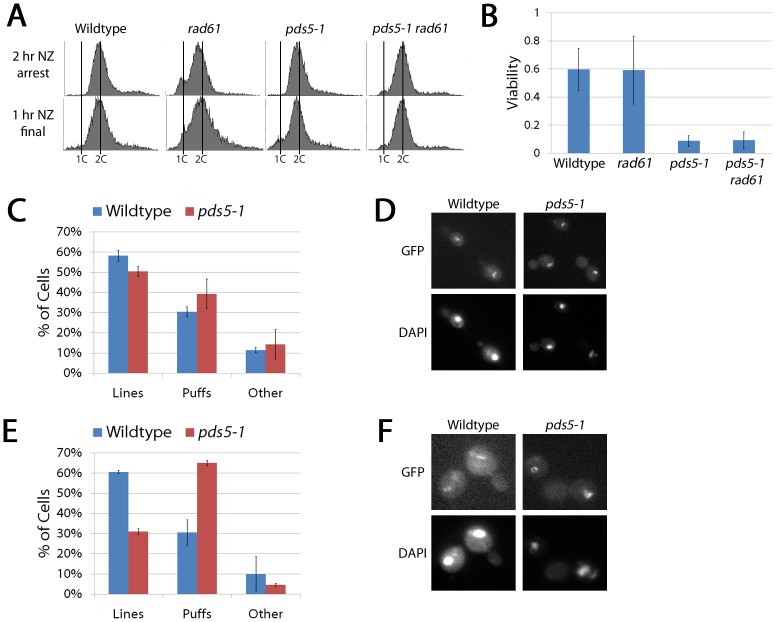
Pds5 is not required to maintain condensation during an extended pre-anaphase arrest. (A) DNA content of wildtype cells and *rad61* and *pds5-1* single mutant cells and *pds5-1 rad61* double mutant cells as described in Figure 1A. (B) Percent viability of wildtype cells and *rad61* and *pds5-1* single mutant cells and *pds5-1 rad61* double mutant cells following the regimen described in Figure 1A. (C) Percent of wildtype and *pds5-1* mutant cells showing condensed (Lines) and uncondensed rDNA (Puffs) rDNA structures following regimen described in Figure 1A. (D) Micrographs of wildtype and *pds5-1* mutant cells highlight rDNA structure through Net1-GFP detection (GFP) and DNA (DAPI). Pds5 inactivation specifically during S-phase impacts chromosome condensation. Wildtype and *pds5-1* mutant cells were synchronized in G1 (alpha factor arrest) at permissive temperature then released to a restrictive temperature and synchronized in pre-anaphase (nocodazole arrest) prior to microscopic analysis. (E) Percent of wildtype and *pds5-1* mutant cells that exhibit either condensed (Lines) or decondensed (Puffs) rDNA structures. (F) Micrographs of wildtype and *pds5-1* mutant cells reveal changes in rDNA architecture.

Rad61/WAPL binds Pds5 and is implicated in regulating cohesin dynamics [21], [24], [25]. While cohesin binding to DNA is not globally decreased upon Pds5 inactivation during mitosis (Figures 2–4), we decided to test whether deletion of *RAD61/WAPL* might rescue *pds5-1* mutant cell inviability. Log phase wildtype, *pds5-1* and *rad61* single mutants, and *pds5-1 rad61* double mutant cells were synchronized in pre-anaphase and then shifted to the non-permissive temperature while retaining the mitotic arrest (Figure 6A). Normalized cell numbers from the resulting cultures were then plated onto rich medium and assessed for cell viability as described above. Both wildtype and *rad61/wapl* mutant cells exhibit fairly robust levels of cell viability (approximately 60%). In contrast, *pds5-1* mutant cells exhibit a markedly low level of cell viability (8%), confirming prior results (Figures 6B and 1). Importantly, *pds5-1 rad61* double mutant cells exhibit a nearly identical low level of cell viability (9%) as *pds5-1* single mutant cells (Figure 6B). Thus, loss of cell viability upon Pds5 inactivation during mitosis is not due to a Rad61/WAPL-dependent increase in cohesin dynamics.

*RAD61* deletion is known to bypass the lethality of *eco1/ctf7* mutant cells, not by rescuing the cohesion defect but rather by rescuing the condensation defect that occurs upon Eco1/Ctf7 inactivation [12], [24]–[26]. Since deletion of *RAD61/WAPL* from *pds5-1* mutant cells failed to increase cell viability when shifted to the restrictive temperature during a mitotic arrest, we hypothesized that *pds5-1* mutant cells are not deficient in maintaining chromosome condensation, even though prior evidence documented that Pds5 inactivation starting from G1 does produce condensation defects [31]. Net1-GFP is well-established as a tool suitable for detecting cohesin-dependent changes in rDNA chromatin architecture [12], [23], [31], [35], [50]. Wildtype and *pds5-1* mutant cells expressing Net1-GFP were arrested in mitosis at the permissive temperature and subsequently shifted to the restrictive temperature while maintaining the mitotic arrest. We then quantified Net1-GFP as forming either linear/loop structures (in which the rDNA loci are clearly distinguishable as well-defined axial elements which often form a tight loop) or puff-like structures in which no clear axial resolution is discernable [12], [23], [31], [35]. The results show that mitotic wildtype and *pds5-1* mutant cells both contain similar levels of condensed "linear" rDNA structures (58% to 50% respectively) that exceeds the level of uncondensed "puffed" structures (30% to 39% respectively) (Figure 6C,D).

To confirm previous reports that *pds5-1* mutant cells exhibit condensation defects when shifted to the restrictive temperature prior to S-phase, we repeated our analysis but now arresting wildtype and *pds5-1* mutant cells in late G1 at a permissive temperature in medium supplemented with alpha-factor and then releasing those cultures to the restrictive temperature in fresh medium supplemented with nocodazole to synchronize cells in pre-anaphase. Results from the Net1-GFP analyses reveal that *pds5-1* mutant cells exhibit a significant condensation defect (65% puffed structures) when compared to wildtype (31%) (Figure 6E,F). In combination, these results confirm the condensation defect shown previously when Pds5 is inactivated during cohesion establishment [31] and reveal for the first time that, once established, Pds5 plays only a marginal role in maintaining chromosome condensation. Herein, we refer to this as a *Condensation Establishment Reaction* that depends on Pds5 and that occurs concomitantly with cohesion establishment.

### Pds5 role in cohesin loading during S-phase is separate from its essential role in cohesion establishment

**Figure 7:**
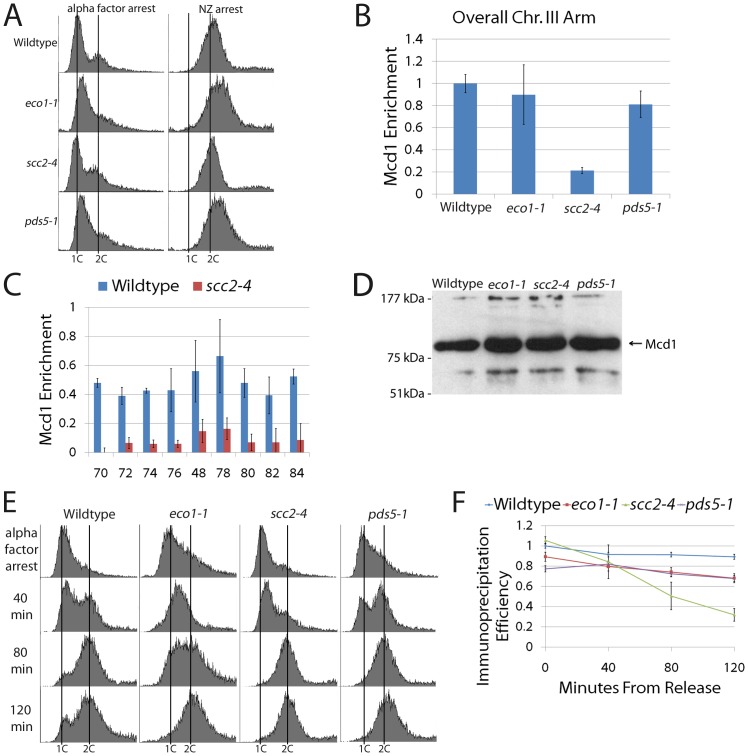
Pds5 is not essential for cohesin enrichment onto DNA during cohesion establishment. (A) DNA content of wildtype cells and *eco1-1*, *scc2-4* and *pds5-1* mutant cells synchronized in G1 (alpha factor arrest) at permissive temperature and then shifted to the restrictive temperature in fresh media supplemented with nocodazole (NZ) to synchronize cells in pre-anaphase. (B) Overall Mcd1 enrichment on chromosome arm sites for wildtype cells and *eco1-1*, *scc2-4* and *pds5-1* single mutant cells treated as described above to obtain pre-anaphase synchrony. Mcd1 levels in wildtype cells normalized to 1. (C) Validation of both *scc2* mutant strains and each of the nine pericentromeric primer sites in which Scc2 inactivation results in substantially reduced Mcd1 enrichment to DNA. (D) Western blot analyses revealing that Mcd1 is present in whole cell extracts obtained from wildtype cells and *eco1-1*, *scc2-4* and *pds5-1* mutant cells. (E and F) Kinetic ChIP analyses of wildtype, *eco1-1*, *scc2-4* and *pds5-1* mutant cell aliquots harvested at 40 minute increments starting from the G1 release and processed for ChIP.

Does Pds5 function during S-phase, when cohesion is first established, differ from its role during mitosis when cohesion is maintained? Numerous studies document a role for Pds5 during cohesion establishment [24], [25], [33], [34], [36]–[38], [51] and at least one study suggests that Pds5 is critical for cohesin enrichment to DNA during S-phase [32]. To address this latter possibility, log phase wildtype, *eco1-1, scc2-4*, and *pds5-1* mutant cells, all expressing Mcd1-3HA as the sole source of Mcd1, were synchronized in G1 and then released to the non-permissive temperature in fresh media supplemented with nocodazole to arrest cells pre-anaphase (Figure 7A). The resulting mitotic cells were then harvested and ChIPs performed to assess the level of Mcd1 enrichment onto DNA at CAR arm sites. Quantification of ChIPs averaged from 3 independent experiments document that wildtype cells retain high levels of Mcd1 enrichment to DNA (Figure 7B). As expected, *scc2-4* mutant cells instead exhibit a massive reduction in Mcd1 enrichment to chromatin (about 20% compared to wildtype cells) whereas *eco1-1* mutant cells retain high levels of chromatin-bound cohesins (Figure 7B), despite a regimen that produces significant cohesion defects [10], [12], [13]. This latter ‘*cohesin without cohesion*’ phenotype typifies establishment mutations [52]. Importantly, *pds5-1* mutant cells retain Mcd1 enrichment onto DNA (about 80% compared to wildtype and about 90% compared to *eco1-1* mutant cells), recapitulating the establishment phenotype (Figure 7B).

To further validate both the *scc2-4* mutant cell control strain and the pericentromeric CAR sites employed throughout this study, we performed ChIP using the primer pairs previously analyzed (Figure 4A). As before, cells synchronized in G1 at the permissive temperature were released to the restrictive temperature in fresh medium supplemented with nocodazole to arrest cells pre-anaphase. Results from ChIP analyses reveal that cohesin enrichment to DNA is substantially reduced along the entire pericentromeric DNA region in *scc2-4* mutant cells (Figure 7C), consistent with the loss of cohesin enrichment along the chromosome arm (Figure 7B). Western blot analyses confirmed that Mcd1 was present in all strains, obviating the model that cohesion loss occurs predominantly through premature Mcd1 proteolysis (Figure 7D). We further tested the possibility that cohesin dissociated early during the cell cycle (S or G2 phases), producing cohesion loss, and that the cohesin detected by ChiP was redeposited late in the cell cycle during pre-anaphase. Wildtype and *eco1-1*, *scc2-4* and *pds5-1* single mutant cells synchronized in G1 at the permissive temperature were released at the restrictive temperature into fresh medium supplemented with nocodazole. In this case, however, culture samples were harvested at 40 minute time increments to map cell cycle progression concomitantly with Mcd1 enrichment onto DNA (Figure 7E). ChIP analyses reveals that, except for *scc2-4* mutant cells, all other strains retain cohesin enrichment to DNA throughout the time course of the experiment (Figure 7F). These results exclude the possibility that cohesin was lost early in the cell cycle and reloaded during the mitotic arrest. In combination, these studies document that the essential role for Pds5 during cohesion establishment is independent of cohesin enrichment onto chromatin.

### Pds5 is not required for Smc3 acetylation during cohesion establishment

**Figure 8:**
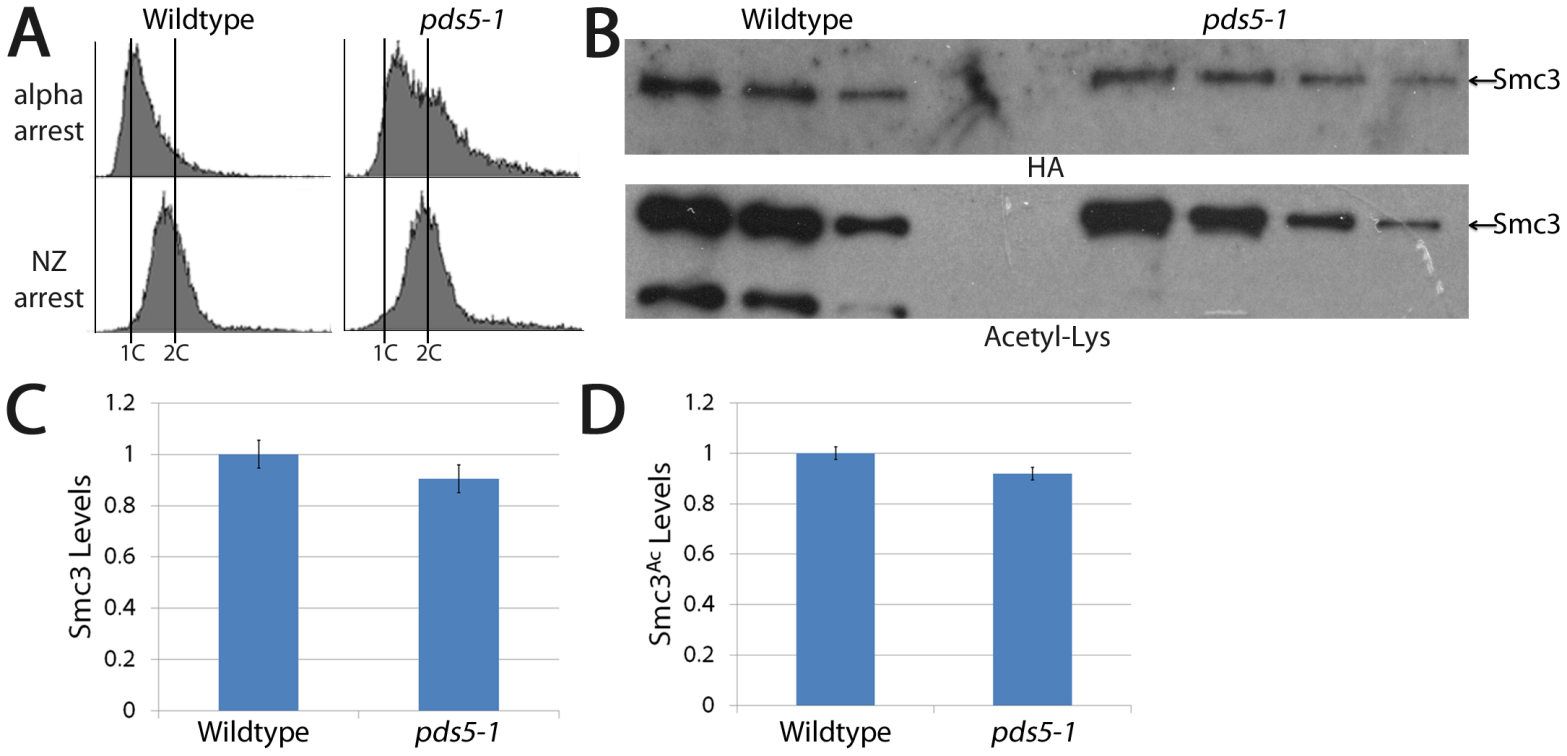
Establishment of sister chromatid cohesion during S-phase is abrogated by loss of Pds5 despite normal levels of Smc3 and Smc3 acetylation. (A) DNA content of wildtype and *pds5-1* mutant cells synchronized in pre-anaphase as described in Figure 7A. (B) Dilution series of Smc3 immunoprecipitated from wildtype and *pds5-1* mutant cells revealing similar levels of both total Smc3 protein (HA) and acetylated (Acetyl-Lys) Smc3. (C-D) Quantification of total Smc3 protein (left) and Smc3 acetylation levels (right) in wildtype (normalized to 1) and *pds5-1* mutant cells.

Our finding that cohesin acetylation is retained upon Pds5 inactivation during mitosis does not exclude the possibility that Pds5 plays a key role in cohesin acetylation during S-phase. To test this possibility, log phase wildtype and *pds5-1* strains expressing HA-tagged Smc3 were synchronized in G1, then released at the non-permissive temperature into fresh medium containing nocodazole (Figure 8A). Normalized cell densities were lysed, incubated with anti-HA coupled affinity matrix and the beads washed to remove unbound or weakly associated proteins. Smc3 protein was eluted from the beads and assayed by Western blot to assess both total Smc3 levels and extent of Smc3 acetylation. As before, we performed a dilution series to confirm that sample concentrations fell within the linear range of Smc3 and acetylated Smc3 signal detections (Figure 8B). Western blot results show that cells that progress through S-phase in the absence of Pds5 contain 90% of total Smc3 protein levels compared to wildtype cells (Figure 8C). Moreover, Pds5-deficient cells contain over 90% of acetylated Smc3 compared to wildtype cells (Figure 8D). Thus, the essential role of Pds5 during S-phase occurs independent of Smc3 acetylation.

## Discussion

Prior studies revealed that Pds5 exerts many functions throughout the cell cycle: promoting both cohesin deposition and cohesion establishment during S-phase, inhibiting cohesin deacetylation prior to mitotic exit, and regulating cohesin dynamics [21], [25], [31]–[38], [53]–[55]. One of the major revelations of the current study is that the essential role of Pds5 in maintaining cohesion during mitosis is not necessarily dependent on any of these activities – even if various *pds5* alleles exhibit such defects. Notwithstanding, Pds5 inactivation during mitosis clearly results in cell inviability and premature separation of sister chromatids, despite the retention of cohesins to both chromosome arm and pericentromeric CAR sites. We note recent supporting evidence that cohesion loss during mitosis can occur despite cohesin retention onto sister chromatids, although that study focused primarily on establishment reactions [34]. Our results further document that *pds5-1* mutant cells retain Smc3 acetylation – negating the possibility that this population of cohesin is newly deposited. The inability to detect DNA damage in *pds5* mutant cells reported here and previously, and that Eco1/Ctf7 acetylates Mcd1 (not Smc3) in response to DNA damage [34], [47], further support the assertion that the acetylated Smc3 detected in the current study is retained from Eco1/Ctf7-dependent S-phase activity. Finally, we found no evidence of Smc3 de-acetylation sufficient to account for the loss of cohesion or that cohesion loss occurs through an increase in Rad61-dependent cohesion dynamics. In combination, these findings negate prior models that the essential role of Pds5 is to either prevent Hos1-dependent de-acetylation of Smc3 or preclude Rad61 destabilization of cohesins [24], [25], [36]. Importantly, we also provide novel evidence that Pds5 plays a greatly diminished role in maintaining chromosome condensation during mitosis once it is established during S-phase. While our results do not preclude roles for Pds5 in cohesin enrichment onto DNA, cohesin acetylation/de-acetylation, altering cohesin dynamics or chromatin architecture - activities all attributed to Pds5 based on analyses of separation-of-function alleles [24], [25], [31], [32], [35], our results are clear in revealing that these reported roles are not the essential mechanism through which Pds5 maintains cohesion during mitosis.

**Figure 9:**
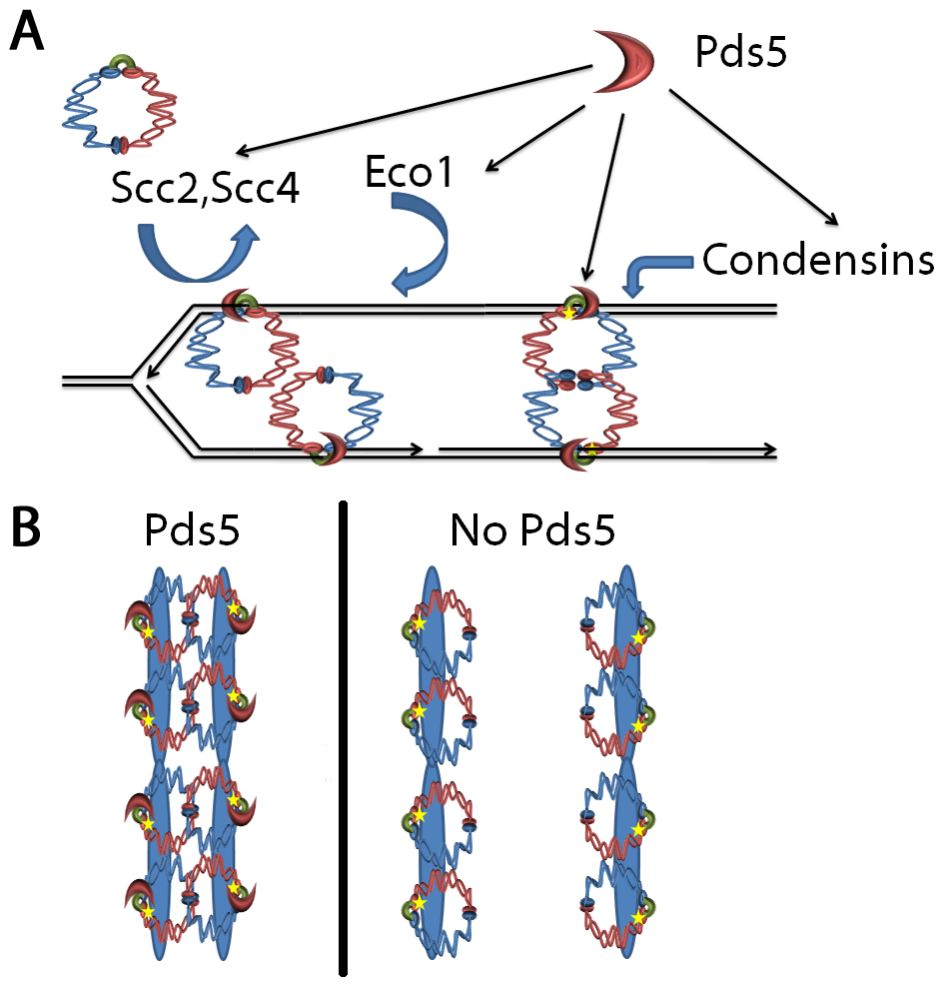
Proposed model of cohesin architecture and Pds5 function. (A) Scc2,Scc4-dependent cohesin loading during S-phase onto nascent sister chromatids is coordinated with Eco1-dependent Smc3 acetylation, leading to stable cohesin-cohesin interactions. Many cohesin structures are possible; shown is one model that reflects recent advances in SMC-like crystal structure studies through which chromatin is captured between SMC head domains and an Mcd1 cap complex [1]. Note the role of Pds5 and Eco1-dependent Smc3 acetylation in regulating hinge-hinge interactions and additional roles for Pds5 in establishing condensation and transcription regulation (not shown). (B) Summary of results that, upon Pds5 inactivation during mitosis, sister chromatid cohesion is lost despite retention of cohesin to DNA and Smc3 acetylation.

Pds5 inactivation during mitosis results in cell death and loss of sister chromatid cohesion, even while both cohesin enrichment and cohesin acetylation are retained. What then, is the role of Pds5 in maintaining cohesion during mitosis and what can we infer about the mechanism through which sister chromatids remain tethered together during mitosis? We initiated the current study to test a presiding model that both sisters reside within a single cohesin ring (*one-ring two-sister chromatids embrace* model). Based on this model, cohesin loss upon Pds5 inactivation must be mediated through cohesin ring opening and dissociation from one or both sisters - either through increased cohesin dynamics (Rad61/WAPL), loss of Smc3 acetylation (Hos1), or cohesin degradation. The second revelation of the current study is that each of the predictions failed to be borne out by the data. We thus favor instead a preceding model that each sister is individually decorated with cohesins [12], [19]. Do cohesin rings entrap each sister chromatid? While cohesin rings remain a popular model, we note evidence of Mcd1 dimerization, analogous to Mre11 dimers in MRN complexes that contain the SMC-like Rad50 protein, consistent with a model that each sister chromatid may be held between SMC heads and an Mcd1 capping complex [1], [56]–[58]. The intimate positioning of DNA between Smc1,3 ATPase heads and an Mcd1 capping structure, as opposed to DNA passively retained within a cohesin ring lumen distal from these active sites, provides a satisfying model for not only the regulation of cohesion, but also for condensation and DNA repair properties of SMC-type complexes (Figure 9). Regardless of the cohesin structure through which cohesins remain associated to DNA, a one cohesin per sister model allows for cohesion loss through cohesin-cohesin dissociation - even while both sisters retain cohesin binding and Smc3 acetylation (Figure 9). We further hypothesize that chromatin looping in *cis*, which brings enhancer/promoter elements into close apposition for transcription, is similarly stabilized by cohesin-cohesin assemblies [1]. Note that this functionally conserved one cohesin per sister (or locus) model is supported by numerous findings that cohesion loss can occur despite full cohesin enrichment and acetylation [10], [12], [13], [18], [31], [34], [35], [53]. In light of our current study, prior results that removing the de-acetylase Hos1 fails to significantly recover cohesion defects in *pds5* mutant cells are well accommodated [36].

In many respects, the long-lived popularity of a one-ring two-sister chromatid embrace model is surprising. Early studies of both Eco1/Ctf7 and Pds5 provided ample proof-of-principal that cohesin deposition and subsequent DNA replication through the ring, mainstays of the one-ring two-sister chromatid embrace model, were inadequate to engender sister chromatid cohesion [10], [12], [31]. Recent analyses of Chl1 DNA helicase as promoting Scc2 recruitment to chromatin during S-phase, coupled with a prior study that mapped Scc2 function to S-phase, confirm that those cohesins loaded during G1 do not participate in cohesion [2], [8]. Instead, it is clear that both cohesion deposition and Eco1/Ctf7-dependent cohesin modification occur in the wake of the DNA replication fork [1]. The finding that histone modifications are central to cohesion maintenance, and that cohesin is retained in H2A.Z mutant cells that exhibit cohesion defects, provides compelling evidence for the model in which cohesin deposition and modification occur in concert with chromatin-assembly reactions [1], [18]. Gartenberg and colleagues demonstrated that cohesion between sister chromatids can be mediated by different complexes (for instance, Sir2 complex association with cohesins), in which each resides on a sister chromatid and linked together [59]. The apparent bias in favoring a one-ring two-sister chromatid embrace is perpetuated by the erroneous notion that there is a difference in capabilities (‘strong’ versus ‘weak’) among cohesin structures [60]. By definition, every model must include as a founding principal that the protein associations required for cohesion are sufficient to withstand mitotic forces – regardless of architecture.

What is the consequence of a one cohesin per sister chromatid model beyond cohesion maintenance? We are particularly intrigued by the findings that, while cohesins are maintained at most CARs upon Pds5 inactivation, some regions show a modest decrease in cohesins while other regions show a modest increase in cohesin enrichment compared to wildtype. From this, we propose that cohesins tethered together to maintain cohesion are relatively restricted from migrating along DNA. Upon cohesion inactivation, our data suggests that each cohesin complex is able to diffuse along DNA – some cohesin towards CAR sites (resulting in increased enrichment) and some away from CAR sites (resulting in decreased enrichment). This implies that Pds5 not only maintains the tethering together of sister chromatids, but also ensures cohesin enrichment at specific locations on DNA, possibly to ensure transcriptional identity between sisters. Currently, it remains unknown whether the cohesin-diffusion phenomenon posited here requires transcription [55] or occurs independent of the presumptive transcription-driven migration of cohesin along DNA. We note, however, that a transcriptional mechanism of cohesin migration does not appear to be a conserved feature – even in yeast [61]–[65]. Thus, the emerging model of cohesin-cohesin interactions also contradicts the speculative view that a single cohesin ring stabilizes DNA looping in *cis* during transcription [65], [66]. Notably, *PDS5/APRIN* mutations are implicated in both cancer progression and birth defects [39]–[42] – the latter of which appears attributable to transcription dysregulation [39], [42]. Thus, insights into novel mechanisms through which Pds5 inactivation might enable each cohesin complex to exert different transcriptional effects – but independent of global cohesion defects as seen in Cornelia de Lange [40], [41], [65], may prove to be of clinical interest.

During final revision of the current study, an article published by D'Ambrosio and Lavoie [67] reported both that Mcd1-6HIS-3FLAG is reduced in whole cell extracts obtained from *pds5-1* mutant cells. Our results extends these findings to suggest that it is the soluble pool of cohesin that is predominantly targeted for degradation with the chromatin-bound cohesins appearing relatively refractile to Pds5 inactivation during mitosis. D'Ambrosio and Lavoie also reported that binding to chromatin was reduced in *pds5-1* mutant cells, relative to wildtype [67]. The decrease reported for the one site in the D'Ambrosio and Lavoie study, however, does not necessarily conflict with our results in that we exploit different epitope tags and quantify cohesin binding at 13 sites different from the site reported in the D'Ambrosio study. As noted above, one mechanism consistent with this loci-specific variability is diffusional mobility upon cohesin-cohesin de-anchoring through loss of cohesion. A more interesting explanation, however, is that the role of cohesins in a particular function (cohesion, condensation, DNA repair, silencing or transcription) that occur within discrete chromatin contexts are uniquely sensitive to Pds5 alterations. In the broader context, these and other studies bring to light an amazing range, revealed within individual *pds5* alleles, through which Pds5 functions in cohesin loading, cohesion establishment, cohesion maintenance and chromosome condensation [24], [25], [31], [32], [35], [36]. It is not, however, the phenotypic range of *pds5* mutant cells that impacts models of cohesion maintenance, but rather the identification of allelic (*pds5-1*) inactivation that results in both cell inviability and loss of cohesion but in the relative absence of either cohesin loss or Smc3 de-acetylation. A growing body of evidence supports both the cohesin without cohesion phenotype and allele-specific roles of Pds5 in cohesin retention [10], [12], [13], [31], [34], [35], [53], [59], [68]. The simplest model emanating from these findings is that cohesin complexes associate with each sister chromatid as they emerge from behind the DNA replication fork and that cohesion is maintained through cohesin-cohesin interactions.

## Materials and Methods

### Genetic manipulations for epitope-tagging or gene deletion

Deletion of *RAD61* was performed and independently confirmed as previously described [69]. C-terminal tags were engineered as previously described [70] within endogenous encoding genes *MCD1*and *SMC3* (Mcd1-3HA and Smc3-3HA). Primers used for *MCD1* are (forward primer) 5'-AGAAGCATTCGGAAATATTAAAATAGACGCCAAACCTGCACTATTTGAAAGGTTTATCAATGCTCGGATCCCCGGGTTAATTAA-3' and (reverse primer) 5'-AAGAAGATTGTTTGGCCTGGAAAACTTTCTAGACGTGGCTTTATTACCAGGGTTGTGTAAGTTAGAATTCGAGCTCGTTTAAAC-3'. Primers used for *SMC3* are (forward) 5'-GGTTATTGAGGTCAATAGAGAAGAAGCAATCGGATTCATTAGAGGTAGCAATAAATTCGCTGAACGGATCCCCGGGTTAATTAA-3' and (reverse) 5'-TTTAGGTAAGAAGAAGCCAAGTGGTGGATTTGCATCATTAATAAAAGATATTTCAAGAAAAGAATTCGAGCTCGTTTAAAC-3'. Integrations were confirmed by PCR using primers 5'-CTGGCGAATTACTTCAAGGCA-3' (*MCD1*) and 5'-GCGGCTCGAGATTCTTGTTCAATCGTTGTAACTCAGC-3' (*SMC3*) in combination with 5'-AACTGCATGGAGATGAGTGGT-3' (*TRP1*). Epitope-tagged protein production was confirmed by Western blot.

### Synchronization of Log Phase Cells and Flow Cytometry

Synchronization of yeast cultures and assessment of DNA contents by flow cytometry were performed as previously described [71]. All strains and genotypes are listed in Table 1.

**Table 1:** Yeast strains used in this study.

Strain	Genotype	Reference
YMM 616	*MATa ade2-1 his3-11,15 leu2-3,112 trp1-1 ura3-1 can1-100*	This study
YMM 843	*MATa ade2-1 his3-11,15 leu2-3,112 trp1-1 ura3-1 can1-100 pds5-1*	Maradeo et al 2010
K6566	*MATa ade2-1 his3-11,15 leu2-3,112 trp1-1 ura3-1 can1-100 MCD1∶18Myc::TRP1*	Michaelis et al 1997
YMM324	*MATa ade2-1 his3-11,15 leu2-3,112 trp1-1 ura3-1 CTF7:ADE2 URA3:tetO LEU2:tetR-GFP TRP1:PDS1-MYC13*	This study
KT034	*MATa ade2-1 his3-11,15 leu2-3,112 trp1-1 ura3-1 CTF7:ADE2 URA3:tetO LEU2:tetR-GFP TRP1:PDS1-MYC13 pds5-1*	This study
KT039	*MATa ade2-1 his3-11,15 leu2-3,112 trp1-1 ura3-1 can1-100 pds5-1 MCD1∶18Myc::TRP1*	This study
KT046	*MATa ade2-1 his3-11,15 leu2-3,112 trp1-1 ura3-1 can1-100 MCD1∶3HA::TRP1*	This study
KT048	*MATa ade2-1 his3-11,15 leu2-3,112 trp1-1 ura3-1 can1-100 scc2-4 MCD1∶3HA:TRP1*	This study
KT047	*MATa ade2-1 his3-11,15 leu2-3,112 trp1-1 ura3-1 can1-100 eco1-1:ADE2 MCD1∶3HA:TRP1*	This study
KT051	*MATa ade2-1 his3-11,15 leu2-3,112 trp1-1 ura3-1 can1-100 pds5-1 MCD1∶3HA::TRP1*	This study
KT052	*MATa ade2-1 his3-11,15 leu2-3,112 trp1-1 ura3-1 can1-100 SMC3∶3HA::TRP1*	This study
KT053	MATa *ade2-1 his3-11,15 leu2-3,112 trp1-1 ura3-1 can1-100 pds5-1 SMC3∶3HA:TRP1*	This study
KT059	*MATa ade2-1 his3-11,15 leu2-3,112 trp1-1 ura3-1 can1-100 rad61Δ::URA3*	This study
KT060	*MATa ade2-1 his3-11,15 leu2-3,112 trp1-1 ura3-1 can1-100 pds5-1 rad61Δ::URA3*	This study
KT062	*MATa ade2-1 his3-11,15 leu2-3,112 trp1-1 ura3-1 can1-100 NET1:GFP:TRP1*	This study
KT064	*MATa ade2-1 his3-11,15 leu2-3,112 trp1-1 ura3-1 can1-100 pds5-1 NET1:GFP:TRP1*	This study

All strains are in W303 unless otherwise specified.

### Viability Assay

Cultures were grown in high nutrient YPD media to an OD_600_ of 0.2, synchronized in G1 (alpha factor) or pre-anaphase (nocodazole) at permissive temperature (23°C) for 3 hours, shifted to non-permissive temperature (37°) for 1–2 hours in the presence of fresh media supplemented with either alpha factor or nocodazole and then placed on high nutrient YPD media for 16 hour at 23°C. Viability was scored by the ability to form microcolonies (colonies with over 30 cells).

### Cohesion Assay

Cohesion assays were performed as previously described with the following modifications [69], [71]. Cells were normalized to 0.1–0.2 OD_600_ and incubated in rich medium supplemented with nocodazole for 2.5 hours at 23°C to synchronize in pre-anaphase. Cells were then shifted to 37°C for 1 hour in the presence of fresh media supplemented with nocodazole to maintain the mitotic arrest. Cell aliquots were harvested at indicated time points, incubated in paraformaldehyde fixation solution, incubated in zymolyase to digest the cell wall and then adhered to a glass slide prior to microscopic analyses. Only large budded cells and in which both DNA (DAPI) and Pds1 (A-14 anti-MYC (Santa Cruz Biotechnology followed by goat anti-rabbit Alexa 568 (Molecular Probes, Inc., Eugene, OR)) were co-incident were analyzed to ensure quantification of pre-anaphase cells. Cells in which sister chromatids remained tightly tethered together appeared to contain a single spot (2 GFP signals so closely apposed as to be indistinguishable from 1 GFP signal) which cells in which sister chromatids prematurely separated were readily apparent by containing 2 GFP spots. Scored cells for cohesion (one versus two GFP spots) were large budded and contained coincident DAPI and Pds1 staining. Cells images captured using a Nikon Eclipse E800 microscope equipped with a cooled CD camera (Coolsnapfx, Photometrics) and IPLab software (Scanolytics). Cohesion analyses were repeated three times and a total of at least 300 cells counted.

### Chromatin Immunoprecipitation and Quantitative PCR

ChIP was performed as previously described [5], [8], with the following modifications. Log phase growth yeast (minimum of 0.6 OD_600_) grown in high nutrient YPD broth were synchronized in either G1 (alpha factor) or pre-anaphase (nocodazole) for 3 hours, shifted to the non-permissive temperature of 37°C for 2 hours and then fixed in 1% formaldehyde for 2 hours. Mcd1 enrichment was obtained by incubating extracts with EZ-view Red Anti-C-Myc affinity matrix (Sigma) or EZ-View Red Anti-HA affinity matrix (Sigma) overnight at 4°C. Beads were collected by centrifugation, washed with TSE-150 (0.1% SDS; 1% Triton X-100; 2 mM EDTA; 150 mM NaCl; 20 mM Tris-Cl pH 8.1) and LiCl/Detergent Wash (0.25 M LiCl; 1% IPEGAL; 1% DOC; 1 mM EDTA; 10 mM Tris-Cl pH 8.1) and the remaining bead-bound proteins harvested using 1%SDS; 0.1 M NaHCO3. DNA-protein crosslinks were reversed in 5 M NaCl. DNA precipitation from the resulting lysate was performed by overnight incubation at −20°C in ethanol. Precipitates were extracted in series using 25∶24∶1 phenol:chloroform:isoamylalcohol and pure chloroform prior to reprecipitation of DNA overnight at −20°C in ethanol. DNA was resuspended in water and analyzed by PCR using CAR site primers previously described [5], [72]. PCR products were resolved using 1% agarose gels, and histograms of pixel densities quantified in Photoshop. Mcd1 enrichment was calculated as the ratio of pull down (ChIP) minus background (obtained using a GST only control) all over total chromatin minus background (obtained using a GST only control).

For quantitative-PCR (qPRC), DNA collected obtained following the above ChIP procedure was measured for Ct values using Rotor-gene (Corbett) and E-values calculated for each individual primer sets. Immunoprecipitation efficiency was determined using the following equation: E-value∧((Ct_Total_ - Ct_ChIP_)-(Ct_Total_- Ct_GST[negative]_)), modified from [73]. All E-values fell between 1.8–2.0.

### Chromatin Binding Assay

Chromatin binding assay was performed as previously described with modifications [8]. Briefly, cells were cultured to an OD_600_ of 0.4, arrested in pre-anaphase (nocodazole), pelleted and washed with 1.2 M Sorbitol. Cells were resuspended in CB1 buffer (50 mM Sodium citrate, 1.2 M Sorbitol, 40 mM EDTA, pH 7.4). Cells were spheroblasted, and resuspended in 1.2 M Sorbitol and frozen in liquid nitrogen. Cells were thawed on ice and supplemented with Lysis buffer (500 mM Lithium Acetate, 20 mM MgSO_4_, 200 mM HEPES, pH 7.9), protease inhibitor cocktail (Sigma), and TritonX-100. Lysate was centrifuged at 12,000×g for 15 minutes and supernatant containing soluble fraction and pellet containing chromatin bound fraction were collected and supplemented with 4X Laemelli (Amresco). Whole cell extracts, supernatant, and pellet were resolved by SDS-PAGE and analyzed using c-Myc (9E10) (Santa Cruz), H2B (Santa Cruz), and PGK (Invitrogen).

### Acetylation Assay

C-terminally tagged Smc3 strains were grown to 0.1–0.3 OD_600_, arrested in pre-anaphase (nocodazole), pelleted by centrifugation, resuspended in IPH150 (150 mM NaCl, 50 mM TRIS pH 8, 5 mM EDTA, 0.5% IGEPAL-CA 630 (Sigma), 1 mM DTT, 10 mM Sodium Butyrate, Roche protease inhibitor cocktail, and immediately frozen in liquid nitrogen. Cells were mechanically lysed (Bead-beater, BioSpec) and extracts incubated with EZ-View Red Anti-HA affinity matrix (Sigma). Beads were washed with IPH50 buffer (50 mM NaCl, 50 mM TRIS pH 8, 5 mM EDTA, 0.5% IGEPAL-CA 630 (Sigma), 1 mM DTT, 10 mM Sodium Butyrate, Roche protease inhibitor cocktail), and bead-bound proteins harvested using 4X Laemmli loading buffer (Amresco). Acetylation status was determined by Western blot using 1∶5000 dilution of anti-Acetylated Lysine (Calbiochem) and band densities quantified using Photoshop.

### Condensation Assay

*NET1* was genetically modified as previously described [70] to include DNA sequence that encodes GFP using the following primers: 5'-TTTAGGTAAGAAGAAGAAGCCAAGTGGTGGATTTGCATCATTAATAAAAGATTTCAAGAAAAAACGGATCCCCGGGTTAATTAA-3' and 5'-TGCTTGATTATTTTTTTTTACTAGCTTTCTGTGACGTGTATTCTACTGAGACTTTCTGGTATCAGAATTCGAGCTCGTTTAAAC -3'. Integrations were confirmed by PCR using the following primers: 5'-CGGATTCCAGTTCAGATTCTA-3' and 5'-AACTGCATG?show=[fo]?>GAGATGAGTGGT-3'. Net1-GFP strains were grown to 0.1–0.2 OD_600_, then incubated for 2.5 hours at 23°C in rich YPD medium supplemented with nocodazole or alpha-factor to arrest cells in pre-anaphase or G1 respectively. Cells were shifted to 37°C for 1 hour in fresh media supplemented with nocodazole to maintain the mitotic arrest. Following 4% paraformaldehyde fixation (10 min at 30°C), cells were assayed using an E800 light microscope (Nikon) equipped with a cooled CD camera (Coolsnapfx, Photometrics) and imaging software (IPLab, Scanalytics, Inc).

### DNA damage and Rad53 phosphorylation Assay

Wildtype and *pds5-1* mutant strains were grown to 0.1–0.3 OD_600_, arrested in pre-anaphase (nocodazole), pelleted by centrifugation, resuspended in water, and immediately frozen in liquid nitrogen. Cells were mechanically lysed (Bead-beater, BioSpec) in the presence of Trichloroacetic acid (TCA). The precipitated extracts were then solubilized in 4X Laemelli loading buffer (Amresco) and resolved by SDS-PAGE prior to transfer to PVDF membrane. Western blot analysis to assess the level of Rad53 modification was performed using Goat-anti-Rad53 (Santa Cruz, yC-19), Donkey-anti-Goat HRP secondary and signal detection performed following ECL Prime (GE) manufacturer instructions.
